# High pretreatment plasma Epstein‐Barr virus (EBV) DNA level is a poor prognostic marker in HIV‐associated, EBV‐negative diffuse large B‐cell lymphoma in Malawi

**DOI:** 10.1002/cam4.2710

**Published:** 2019-11-29

**Authors:** Nathan D. Montgomery, Cara Randall, Matthew Painschab, Ryan Seguin, Bongani Kaimila, Edwards Kasonkanji, Takondwa Zuze, Robert Krysiak, Marcia K. Sanders, Avian Elliott, Melissa B. Miller, Coxcilly Kampani, Fred Chimzimu, Maurice Mulenga, Blossom Damania, Tamiwe Tomoka, Yuri Fedoriw, Dirk P. Dittmer, Satish Gopal

**Affiliations:** ^1^ Department of Pathology & Laboratory Medicine University of North Carolina Chapel Hill NC USA; ^2^ Lineberger Comprehensive Cancer Center University of North Carolina Chapel Hill NC USA; ^3^ UNC Project‐Malawi Lilongwe Malawi; ^4^ Department of Medicine Division of Hematology & Oncology University of North Carolina Chapel Hill NC USA; ^5^ Department of Microbiology & Immunology University of North Carolina Chapel Hill NC USA; ^6^ UNC Healthcare Chapel Hill NC USA

**Keywords:** diffuse large B‐cell lymphoma, Epstein‐Barr virus, HIV, prognosis, sub‐Saharan Africa

## Abstract

Plasma Epstein‐Barr virus (EBV) DNA measurement has established prognostic utility in EBV‐driven lymphomas, where it serves as a circulating tumor DNA marker. The value of plasma EBV measurement may be amplified in sub‐Saharan Africa (SSA), where advanced imaging and molecular technologies for risk stratification are not typically available. However, its utility in diffuse large B‐cell lymphoma (DLBCL) is less certain, given that only a subset of DLBCLs are EBV‐positive. To explore this possibility, we measured plasma EBV DNA at diagnosis in a cohort of patients with DLBCL in Malawi. High plasma EBV DNA at diagnosis (≥3.0 log_10_ copies/mL) was associated with decreased overall survival (OS) (*P* = .048). When stratified by HIV status, the prognostic utility of baseline plasma EBV DNA level was restricted to HIV‐positive patients. Unexpectedly, most HIV‐positive patients with high plasma EBV DNA at diagnosis had EBV‐negative lymphomas, as confirmed by multiple methods. Even in these HIV‐positive patients with EBV‐negative DLBCL, high plasma EBV DNA remained associated with shorter OS (*P* = .014). These results suggest that EBV reactivation in nontumor cells is a poor prognostic finding even in HIV‐positive patients with convincingly EBV‐negative DLBCL, extending the potential utility of EBV measurement as a valuable and implementable prognostic marker in SSA.

## Graphical abstract

In a cohort of HIV‐positive patients with diffuse large B‐cell lymphoma (DLBCL) in Malawi, we demonstrate that high plasma Epstein‐Barr virus (EBV) DNA level at diagnosis (≥3.0 log_10_ copies/mL) is associated with decreased overall survival. Notably, even in those HIV‐positive patients with EBV‐negative tumors, high plasma EBV DNA remained associated with shorter survival. These results suggest that EBV reactivation in nontumor cells is a poor prognostic finding in HIV‐positive patients with EBV‐negative DLBCL.

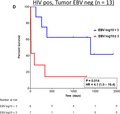

## 1 INTRODUCTION

Epstein‐Barr virus (EBV) is an oncogenic herpesvirus implicated in many lymphomas, as well as some solid tumors.^1^ For a subset of lymphomas, such as endemic forms of Burkitt lymphoma, EBV is present in the tumor cells of nearly all cases.^2^ However, for other lymphomas, such as diffuse large B‐cell lymphoma (DLBCL), both EBV‐negative and EBV‐positive forms of disease are recognized.^3^, ^4^ The likelihood that a patient's DLBCL will be EBV‐associated is primarily related to underlying immune status, with human immunodeficiency virus (HIV) infection, iatrogenic immunosuppression, and advanced age all increasing the likelihood of EBV positivity.^5^

DLBCL is the most common subtype of lymphoma worldwide, and in sub‐Saharan Africa (SSA), the incidence of this and other aggressive B‐cell lymphomas is increasing, primarily due to HIV and population aging.^6^, ^7^, ^8^, ^9^ Prior studies have suggested EBV‐positivity in approximately 80%‐90% of cases of HIV‐associated DLBCL with immunoblastic cytology and approximately 30% of cases with centroblastic cytology.^5^, ^10^ By comparison, less than 10% of DLBCL arising in HIV‐negative individuals is associated with EBV.^5^

Accurate risk stratification of DLBCL remains challenging in low‐income countries, as there is generally no access to positron emission tomography (PET) scans, cell‐of‐origin subtyping assays, or prognostically informative cytogenetic or immunophenotypic studies. While often under‐appreciated, risk stratification is critically important in these settings. Intensive chemotherapy is often associated with treatment‐related morbidity and mortality due to poor supportive care, conferring substantial risks to patients that may be more pronounced than in high‐income countries.

Plasma EBV measurement is a clinically valuable and highly implementable biomarker for many EBV‐associated malignancies.^11^, ^12^, ^13^ We and others have suggested that plasma EBV measurement may be particularly informative in SSA and other low‐income settings, where EBV‐associated lymphomas are common.^14^, ^15^, ^16^ This hypothesis has generally been confirmed for lymphomas that are invariably EBV‐positive, like endemic Burkitt lymphoma and HIV‐associated classic Hodgkin lymphoma.^14^, ^15^, ^17^, ^18^ In such cases, plasma EBV functions as a sensitive circulating tumor DNA (ctDNA) marker, with both prognostic and monitoring utility. Conversely, the value of plasma EBV measurement for DLBCL, which is EBV‐associated in a minority of cases, is less clear. We therefore sought to correlate pretreatment plasma EBV level with survival in a cohort of patients with DLBCL in Malawi.

## 2 METHODS

### 2.1 Study participants

All patients included in this study were enrolled in the Kamuzu Central Hospital (KCH) Lymphoma Study. Details of this study have been described previously^19^, ^20^; but briefly, the KCH Lymphoma Study is an ongoing prospective observational cohort based at a national teaching hospital in Lilongwe, Malawi. KCH is one of two referral centers for cancer care in Malawi, with a catchment area of approximately 9 million people.

For this analysis, all patients were adults (≥18 years) diagnosed with DLBCL between June 1, 2013 and May 31, 2016. All DLBCL diagnoses were confirmed by tissue biopsy, supported by manual immunohistochemistry (IHC) and a weekly clinicopathologic teleconference attended by pathologists and oncologists in the United States and Malawi.^20^ Subsequently, tissue blocks were sent to the University of North Carolina (UNC) at Chapel Hill for diagnostic confirmation and additional ancillary studies, as described previously and below.^20^ In addition to a confirmed diagnosis of DLBCL, the only other inclusion criterion for this study was availability of a pretreatment plasma EBV level (see below).

First‐line chemotherapy for patients with DLBCL in this cohort was CHOP (cyclophosphamide, doxorubicin, vincristine, prednisone), along with concurrent antiretroviral therapy in HIV‐positive patients. All participants were followed until death, or administrative censoring on November 1, 2018, with none lost to follow‐up.

### 2.2 Plasma EBV measurement

Plasma EBV measurements were performed in the UNC Vironomics Core Facility, as previously described.^14^ Briefly, anti‐coagulated plasma was collected prior to initiation of cytotoxic chemotherapy and then stored at −80°C, prior to shipment to UNC. Quantitative plasma EBV measurement was performed using a quantitative real‐time polymerase chain reaction (qPCR) assay with primer pairs designed to amplify a conserved region of the EBV *EBNA3C* gene, corresponding to positions 88933‐89033 of the EBVI reference genome and positions 89374‐89836 of the EBVII reference genome. The linear detection range for this assay is 2.0‐8.0 log_10_ copies/mL, with values <2.0 log_10_ copies/mL reported as “not detected”.

### 2.3 Tumor EBV staining

EBV staining was performed on tissue blocks in the UNC Translational Pathology laboratory by three methods: EBER ISH (Novocastra EBER Probe; Leica Biosystems), LMP1 IHC (Abcam), and EBNA1 IHC (Novus USA). All stains were performed on a Leica Bond Max instrument, according to the manufacturer's instructions, with chromogenic detection via Leica Bond Polymer AP Red Detection Kit for ISH and Leica Bond Intense R Detection Kit supplemented with Novocastra Novolink Polymer Detection System for IHC. Tumors were considered stain positive if at least 50% of the neoplastic cells were stained. Importantly, however, only a single negative case had between 1% and 50% positive cells (patient 25 in Table 3). In all cases, results were compared to appropriate controls, including confirmation of adequate RNA preservation for interpretation of negative EBER ISH (Epstein‐Barr encoded RNA in situ hybridization) stains.

### 2.4 Tumor EBV polymerase chain reaction

DNA was extracted on a Maxwell® 16 MDx instrument (Promega) using 5 × 10 μm scrolls prepared from the diagnostic formalin‐fixed, paraffin embedded tissue block. Then, EBV was detected by a qPCR assay targeting the BamH1W segment of EBV, as previously described.^21^ Cases were considered positive if the fluorescence signal crossed the critical threshold in fewer than 40 cycles.

### 2.5 Statistical analysis

Patient clinical characteristics were compared by Mann‐Whitney U‐Test (for continuous data) or by Fisher Exact Test (categorical data). Survival curves were compared and hazard ratios were calculated by log rank test. All statistical analyses were performed using GraphPad Prism 8.

### 2.6 Informed consent and IRB approval

Informed consent was obtained in English or Chichewa, the two national languages in Malawi. This study was approved by the UNC Institutional Review Board and the Malawi National Health Sciences Research Committee and is registered with http://clinicaltrials.gov under NCT02835911.

## 3 RESULTS

### 3.1 Patients

A pretreatment plasma EBV measurement was available in 44 patients diagnosed with DLBCL during the study period, including 25 HIV‐positive and 19 HIV‐negative individuals. Clinical characteristics of these patients, stratified by HIV status, are shown in Table 1. As in previous descriptions of this cohort,^19^, ^22^ HIV‐positive patients were younger than their HIV‐negative counterparts (45.4 vs 56.2 years, *P* = .03), with similar male predominance in both groups (19:6 vs 12:7, *P = *.51).

**Table 1:** Clinical characteristics and pretreatment plasma EBV DNA level of all DLBCL patients

	All patients (n = 44)	HIV pot (n = 25)	HIV neg (n = 19)	*P* value
Age, median years (range)	47.1 (23.2‐77.4)	45.4 (24.1‐63.2)	56.2 (23.2 ‐77.4)	.03
Male:female	31:13	19:6	12:7	.51
EBV log_10_ copies/mL at diagnosis, median (range)	2.81 (<2.0‐7.11)	2.81 (<2.0‐6.36)	<2.0 (<2.0‐7.11)	.22
Patients with plasma EBV at diagnosis below LOD, n (%)	22 (50)	10 (40)	12 (63)	.22
Patients with EBV log_10_ > 3.0 copies/mL at diagnosis (%)	16 (36)	10 (40)	6 (32)	.75
Tumor EBV positive by EBER in situ hybridization/total (%)	5/42 (11)*	2/23 (9)*	3/19 (19)	.64

Abbreviations: EBER, EBV encoded RNA; EBV, Epstein‐Barr virus; LOD, limit of detection.

* EBER ISH results were uninterpretable in two HIV‐positive lymphomas due to failed RNA preservation.

### 3.2 Pretreatment plasma EBV measurements and survival

Pretreatment plasma EBV DNA level was also similar between HIV‐positive and HIV‐negative patients (median 2.81 log_10_ copies/mL vs < 2.0, *P* = .22, Table 1). In addition, a similar proportion of HIV‐positive and HIV‐negative patients had negative pretreatment plasma EBV DNA studies, falling below the assay's validated limit of detection of 2.0 log_10_ copies/mL (10/25 vs 12/19, *P* = .22, Table 1). Amongst those patients with a positive plasma EBV result, the median pretreatment plasma EBV DNA level was similar in HIV‐positive and HIV‐negative patients (3.91 vs 3.94, *P* = .70, data not shown). Finally, there was also no difference in the fraction of HIV‐positive vs HIV‐negative patients with a “high” pretreatment plasma EBV DNA level, defined here as ≥3.0 log_10_ copies/mL (10/25 vs 6/19, *P* = .75). This “high” threshold was chosen to define a cutpoint robustly above the limit of detection and above the level typically seen in cancer patients with non‐EBV‐associated tumors.^23^

**Figure 1:**
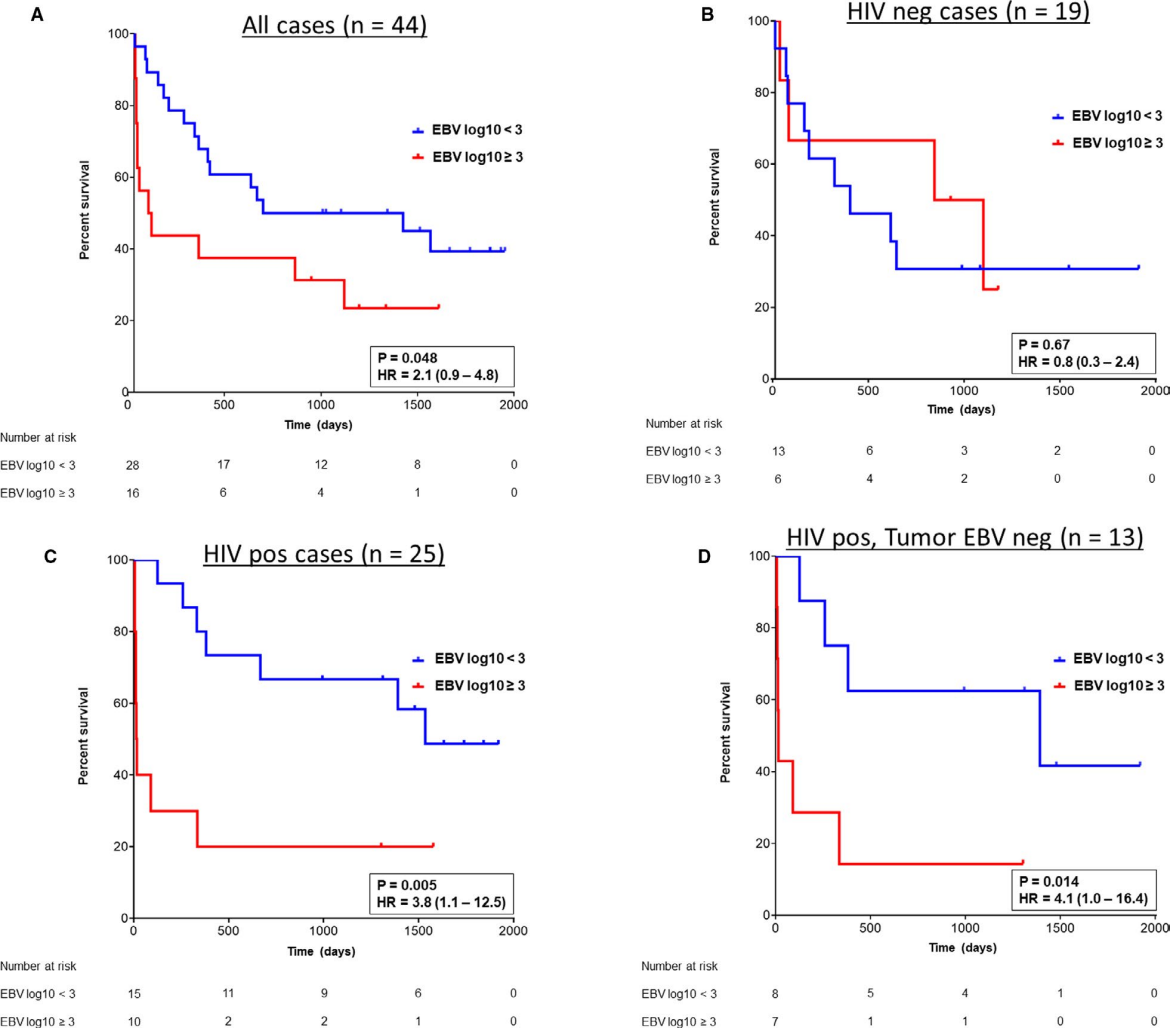
Pretreatment plasma EBV DNA level and overall survival in patients with DLBCL in Malawi. A, All patients with DLBCL; B, HIV‐negative patients with DLBCL; C, HIV‐positive patients with DLBCL; and D, HIV‐positive patients with tumors that are EBV negative by EBER ISH, LMP1 IHC, EBNA1 IHC, and tissue PCR (“quadruple negative.”). For all plots, patients were stratified by pretreatment plasma EBV DNA levels: <3.0 log_10_ copies/ mL (blue) or ≥3.0 log_10_ copies/mL (red). *P* values and hazard ratios with 95% confidence intervals, as determined by log rank test, are shown

Next, we sought to determine whether pretreatment EBV DNA level was correlated with outcomes in Malawian patients with DLBCL. First, we evaluated all study participants, irrespective of HIV status. In the full cohort, high pretreatment plasma EBV DNA was correlated with a significant decrease in overall survival (OS) (*P* = .048 and hazard ratio = 2.1, 95% CI = 0.9‐4.8) (Figure 1A).

In order to determine whether the survival disadvantage of a high pretreatment plasma EBV level might be related to HIV status, HIV‐negative and HIV‐positive patients were analyzed separately. Whereas there was no correlation between pretreatment plasma EBV DNA level and survival in HIV‐negative patients (Figure 1B, *P* = .67), high pretreatment plasma EBV DNA was associated with markedly shorter OS in HIV‐positive patients (Figure 1C, *P* = .005, hazard ratio = 3.8, 95% CI = 1.1‐12.5). Emphasizing the relationship between pretreatment plasma EBV level and survival in HIV‐positive patients, the median OS for patients with high pretreatment plasma EBV DNA was just 16 days, compared to 1534 days for patients with low pretreatment plasma EBV DNA (Table 2, *P* = .005). In addition, all HIV‐positive patients who died in the first 100 days after diagnosis had a pretreatment plasma EBV level ≥3.0 log_10_ copies/mL (Table 2, *P* = .0002), despite having similar CD4 counts at DLBCL diagnosis (198 ± 178 × 10^9^/L in patients dying in <100 days vs 173 ± 154 × 10^9^/L in patients living > 100 days, *P* = .83).

**Table 2:** Clinical characteristics of HIV‐positive patients with DLBCL stratified by pretreatment plasma EBV DNA level

	All HIV‐positive DLBCL				HIV‐positive patients with tumor EBV quadruple negative DLBCL			
	n	EBV log_10_ < 3.0 copies/mL (n = 15)	EBV log_10_ > 3.0 copies/mL (n = 10)	*P* value	n	EBV log_10_ < 3.0 copies/mL (n = 8)	EBV log_10_ > 3.0 copies/mL (n = 7)	*P* value
Median survival (days)	25	1534	16	.005	15	1391	15	.01
Death within 100 d, n (%)	25	0 (0)	7 (70)	.0002	15	0 (0)	5 (33)	.007
Age median years, (range)	25	47.2 (30.3‐63.2)	44.5 (24.1‐53.6)	.16	15	49.3 (30.3‐60.1)	44.6 (32.8‐49.9)	.09
M:F	25	11:4	8:2	1.0	15	6:2	5:2	1
ECOG PS* > *2, n (%)	25	1 (7)	7 (70)	.002	15	1 (13)	5 (71)	.04
Ann Arbor stage IV, n (%)	25	4 (27)	7 (70)	.049	15	2 (25)	4 (57)	.31
IPI > 3, n (%)	24	2 (14)	5 (50)	.09	15	1 (13)	2 (29)	.57
Age adjusted IPI > 3, n (%)	24	1 (7)	4 (40)	.12	15	1 (13)	2 (29)	.57
Lactate dehydrogenase ratio (Patient:ULN), mean ± SD	24	2.0 ± 1.5	4.8 ± 4.4	.02	15	2.5 ± 2.1	5.1 ± 5.2	.18
Lactate dehydrogenase level greater than ULN, n (%)	24	12 (86)	9 (90)	1	15	7 (88)	6 (86)	1
Time since HIV diagnosis, years, mean ± SD	23	3.0 ± 4.6	2.8 ± 3.4	.79	14	2.5 ± 3.4	1.3 ± 2.4	.82
CD4 count, cells × 10^9^/L, mean ± SD	25	197 ± 158	155 ± 161	.20	15	177 ± 110	189 ± 184	.54
HIV viral load at diagnosis, log_10_ copies/µL, mean ± SD	25	2.4 ± 2.4	2.5 ± 2.4	.90	15	1.8 ± 1.7	2.1 ± 2.1	.89
ART naive (<3 mo) at diagnosis, n (%)	25	7 (47)	5 (50)	1.0	15	3 (38)	5 (71)	.31
White blood cell count, 10^9^/L, mean ± SD	25	5.5 ± 15	6.5 ± 2.6	.29	15	6 0 ± 1.2	7.5 ± 2.5	.14

Abbreviations: ART, antiretroviral therapy; DLBCL, diffuse large B‐cell lymphoma; EBV, Epstein‐Barr virus; ECOG, Eastern Cooperative Oncology Group (ECOG); HIV, human immunodeficiency virus; IPI, International Prognostic Index; SD, standard deviations; ULN, upper limit of normal

HIV‐positive patients with high pretreatment plasma EBV were also more likely to have Eastern Cooperative Oncology Group (ECOG) performance status ≥2 and Ann Arbor Stage IV disease (Table 2, *P* = .002 and *P* = .049 respectively). In addition, the ratio of patient lactate dehydrogenase (LDH) to the laboratory's upper limit of normal was higher in HIV‐positive patients with a high pretreatment EBV level, though there was no difference in the fraction of patients with an elevated LDH between HIV‐positive and HIV‐negative groups (Table 2, *P* = .02 and *P* = 1.0, respectively). Both international prognostic index score and age‐adjusted international prognostic index score tended to be higher in HIV‐positive patients with a high pretreatment EBV level, though these differences were not statistically significant (*P* = .09 and 0.12, respectively). Finally, there were no differences in mean age, gender, time since HIV diagnosis, CD4 count, HIV viral load, peripheral white blood cell count, antiretroviral therapy (ART) status if HIV‐positive, or treatment‐related mortality between patients with a high or low pretreatment plasma EBV level (Table 2).

### 3.3 Correlation between tumor EBER ISH and plasma EBV detection

**Figure 2:**
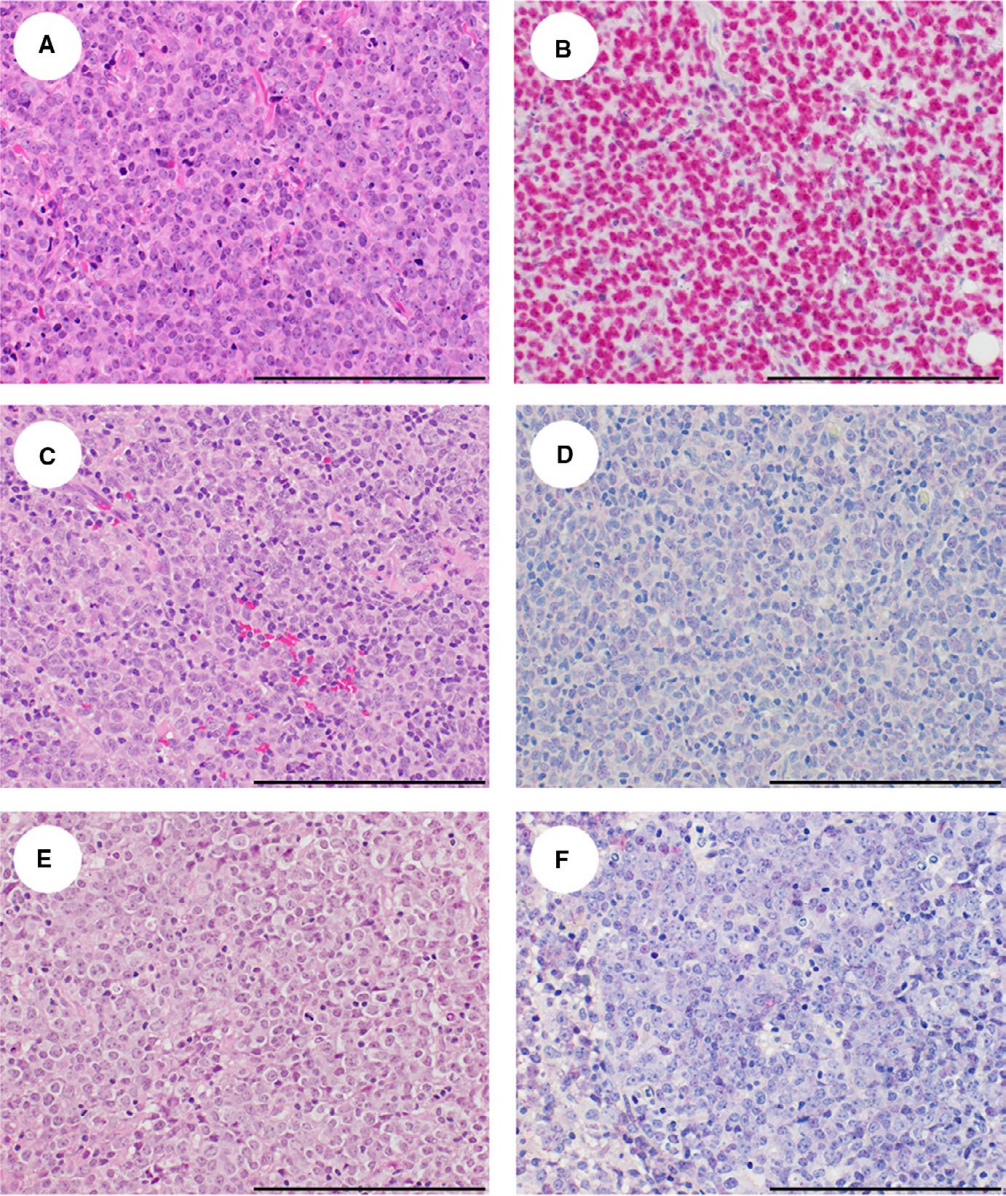
Morphologic findings and EBER stains in representative DLBCL from HIV‐positive patients in Malawi. Representative hematoxylin & eosin stained sections (A,C,E) and EBER in situ hybridization results (B,D,F) of DLBCL from HIV‐positive patients in Malawi. (A,B) Tumor EBER‐positive; (C,D) tumor EBER‐negative with undetectable plasma EBV; (E,F) tumor EBER‐negative with plasma EBV >3.0 log_10_ copies/mL

To determine whether the high plasma EBV detected in some patients correlated with EBV status in the tumor, we evaluated tumor EBV status by EBER ISH. Interpretable EBER ISH results were available on 42/44 patients. Images of representative lymphomas from patients in this cohort are shown in Figure 2. A similar proportion of DLBCLs were EBER ISH positive in HIV‐positive (2/23, 9%) and HIV‐negative (3/19, 16%) patients, with 4/5 positive cases occurring in individuals 54 years of age or older, including all 3 HIV‐negative cases (Table 1, data not shown). Amongst HIV positive cases, there were too few EBER‐positive DLBCLs to make meaningful clinical comparisons between EBER‐positive (n = 2) and EBER‐negative cases (n = 21). However, there was no obvious difference in mean CD4 count between these groups (105 ± 78 × 10^9^/L in EBER‐positive DLBCL vs 179 ± 154 × 10^9^/L in EBER‐negative DLBCL).

Notably, tumor EBER‐positive cases in the cohort (n = 5) were substantially less numerous than cases with a high pretreatment plasma EBV DNA level (n = 16). While 3 of 5 EBER‐positive cases had a pretreatment plasma EBV DNA level ≥ 3.0 log_10_ copies/mL, high pretreatment plasma EBV DNA was also detected in 12/37 tumor EBER‐negative DLBCLs, raising the possibility that plasma EBV DNA may not be tumor‐derived in many DLBCL cases.

To assess other potential sources of plasma EBV DNA in patients with tumor EBER‐negative DLBCL, we also evaluated EBER staining in background, non‐neoplastic lymphocytes. Rare non‐neoplastic EBER‐positive lymphocytes were identified in 6 tumor EBV‐negative DLBCLs, in each case representing <1% of all lymph node cells. Cases with non‐neoplastic EBER‐positive lymphocytes were no more likely than cases without non‐neoplastic EBER‐positive lymphocytes to have a plasma EBV DNA level above the assay's validated limit of detection or ≥3.0 log_10_ copies/mL (*P* = .64 and .66, respectively).

### 3.4 Evaluation of tumor EBV status by orthogonal methods

The existence of rigorous pathology protocols in the KCH Pathology Laboratory, along with confirmation of RNA preservation in 42/44 cases, decreases the likelihood of false negative EBER ISH staining in this cohort. To independently confirm EBV status in the DLBCL, we used two IHC stains, LMP1 (latent membrane protein 1) and EBNA1 (Epstein‐Barr nuclear antigen 1). LMP1 and EBNA1 are co‐expressed with EBER in EBV's type II and III latency programs, which are typically observed in DLBCL.^24^, ^25^ As such, these stains provided an orthogonal method of EBV detection. Insufficient tissue remained for testing in 5 cases, leaving 20 cases of HIV‐positive DLBCL for evaluation, 18 of which had an interpretable EBER stain for comparison. LMP1/EBNA1 results in tumor cells were concordant with EBER ISH in 17/18 cases (95%). The lone discordant case (patient 9) exhibited positive EBER staining but was negative for LMP1/EBNA1.

To further exclude falsely negative EBV detection in HIV‐associated DLBCL cases in our cohort, we next attempted to evaluate EBV status by real‐time qPCR performed on FFPE tissue scrolls (Table 3). Tissue EBV qPCR was concordant with EBER ISH results in 18/18 cases and with LMP1/EBNA1 IHC in 18/20 cases. Both cases with discordant qPCR and LMP1/EBNA1 staining results were qPCR positive and stain negative. One of these cases ( patient 9) was also EBER ISH positive, as mentioned above. In the other, EBER ISH results were uninterpretable due to failed RNA preservation (patient 5).

**Table 3:** Tumor EBV status evaluated by multiple methods

Patient number	Pretreatment plasma EBV ≥3.0	EBER ISH	LMP1 IHC	EBNA1 IHC	Tissue EBV PCR	Tumor EBV quadruple negative	LYM	ISH/IHC concordant	EBER/PCR concordant	
1	NO	NEG	NEG	NEG	NEG	YES	5	YES	YES	1
2	NO	NEG	NEG	NEG	NEG	YES	9	YES	YES	2
3	NO	NEG	QNS	QNS	QNS	N/A	24	QNS	QNS	
4	NO	NEG	NEG	NEG	NEG	YES	34	YES	YES	3
5	NO	Uninterpretable	NEG	NEG	POS	NO	44	Unint.		
6	NO	NEG	NEG	NEG	NEG	YES	86	YES	Equivocal	
7	NO	NEG	QNS	QNS	QNS	N/A	116	QNS	QNS	
8	NO	NEG	QNS	QNS	QNS	N/A	127	QNS	QNS	
9	NO	POS	NEG	NEG	POS	NO	243	NO		
10	NO	NEG	NEG	NEG	NEG	YES	302	YES	YES	4
11	NO	NEG	QNS	QNS	QNS	N/A	15	QNS	QNS	
12	NO	NEG	NEG	NEG	NEG	YES	145	YES	YES	5
13	NO	NEG	NEG	NEG	NEG	YES	159	YES	YES	6
14	NO	NEG	NEG	NEG	NEG	YES	256	YES	YES	7
15	NO	NEG	QNS	QNS	QNS	N/A	81	QNS	QNS	
16	YES	NEG	NEG	NEG	NEG	YES	1	YES	YES	8
17	YES	NEG	NEG	NEG	NEG	YES	192	YES	Equivocal	
18	YES	NEG	NEG	NEG	NEG	YES	204	YES	YES	9
19	YES	NEG	NEG	NEG	NEG	YES	318	YES	YES	10
20	YES	NEG	NEG	NEG	NEG	YES	153	YES	YES	11
21	YES	NEG	NEG	NEG	NEG	YES	202	YES	YES	12
22	YES	NEG	NEG	NEG	NEG	YES	154	YES	YES	13
23	YES	Uninterpretable	POS	POS	POS	NO	121	Unint.		
24	YES	POS	POS	POS	POS	NO	313	YES		
25	YES	NEG (<10%)	NEG (<10%)	NEG (< 10%)	NEG	NO^a^	282	YES	YES	14

Note

“Uninterpretable” indicates that RNA was inadequately preserved for interpretation of EBER stain.

Abbreviations: EBER, Epstein‐Barr encoded RNA; EBNA1, Epstein‐Barr nuclear antigen 1; EBV, Epstein‐Barr virus; IHC, immunohistochemistry; ISH, in situ hybridization; LMP1, latent membrane protein 1; NEG, negative; PCR, polymerase chain reaction; POS, positive; QNS, quantity not sufficient for testing.

a Denotes that the case was classified as not “tumor EBV quadruple negative” due to the small percentage of tumor cells (<10%) positive by EBER, LMP1, and EBNA1 stains.

### 3.5 Pretreatment plasma EBV and survival in HIV‐positive, tumor EBV negative cases

15 of 25 (60%) cases of HIV‐associated DLBCL in this cohort were negative by all four tissue‐based methods used to detect EBV in this study (EBER ISH, LMP1 IHC, EBNA1 IHC, Tissue PCR). We refer to these cases as “tumor EBV‐quadruple‐negative” in order to highlight that there is no evidence to indicate that EBV is present in the neoplastic cells in these lymphomas (Table 3). Nevertheless, 7 of 15 (47%) tumor EBV‐quadruple‐negative lymphomas were from patients with a high pretreatment plasma EBV DNA level, favoring the conclusion that plasma EBV is not tumor‐derived in these individuals. Notably, even in these tumor EBV quadruple negative cases, a high pretreatment plasma EBV DNA level remained associated with shorter OS (Figure 1D) (*P* = .014, hazard ratio = 4.1 with 95%CI 1.0‐16.4). In general, clinical features were similar in tumor EBV‐quadruple‐negative patients compared to the full HIV‐positive cohort (Table 2).

## 4 DISCUSSION

Plasma EBV has been studied intensely for its clinical utility as a predictive or prognostic biomarker in EBV‐driven lymphoma.^26^, ^27^ In SSA, this biomarker has been most extensively evaluated in endemic Burkitt lymphoma and classic Hodgkin lymphoma, both of which are EBV positive in most cases.^14^, ^15^, ^17^, ^18^ Plasma EBV is also a promising lymphoma marker in HIV‐positive patients,^28^ though its utility has been primarily explored in patients with EBV‐positive tumors.

We sought to explore the prognostic utility of plasma EBV in DLBCL in Malawi. This question is of particular relevance for low‐income countries, which have a high incidence of DLBCL, but do not have advanced capabilities like PET scans, that are routinely used for DLBCL risk stratification in high‐income countries. Measuring plasma viral loads is readily implementable in low‐resource settings, especially given that existing laboratory capacity has been developed to support HIV RNA monitoring in public sector ART programs. Although high pretreatment plasma EBV DNA has been reported in patients with EBER‐negative DLBCL,^29^, ^30^ these earlier studies excluded HIV‐positive patients. Here, we extend those observations to HIV‐infected individuals in Malawi and further show that a high pretreatment plasma EBV DNA level is associated with poor overall survival in HIV‐positive, tumor EBV‐negative patients.

More than 90% of the world's population is estimated to be infected with EBV,^31^, ^32^, ^33^, ^34^ and once infected, EBV persists as a latent infection for the remainder of an individual's lifetime.^35^ Latently infected cells are rare in normal individuals, representing fewer than 1 in 10,000 peripheral blood leukocytes.^36^ As a result, EBV DNA is typically undetectable or present only at a very low level in the plasma of normal adults.^27^, ^37^, ^38^ In patients with EBV‐driven malignancies, particularly in the post‐transplant setting, plasma EBV may rise as a ctDNA marker.^39^, ^40^, ^41^, ^42^ In contrast, detection of high plasma EBV among patients in our cohort with EBV tumor‐negative DLBCL strongly suggests that the virus is derived from non‐tumor cells.

However, from this small study, it is not possible to determine underlying mechanisms for poor outcomes in patients with a high pretreatment plasma EBV DNA level. Our findings are reminiscent of cytomegalovirus (CMV) reactivation being associated with worse outcomes in critically ill patients.^43^, ^44^ Analogously, EBV reactivation in nontumor cells of HIV‐positive patients with DLBCL could reflect underlying illness or immune dysfunction not reflected in standard CD4 count assessment. This interpretation is supported by the observation that HIV‐positive patients with high pretreatment plasma EBV DNA levels were more likely to have an ECOG performance status ≥2, with a similar trend persisting in tumor EBV‐quadruple‐negative DLBCL. It is also possible that EBV reactivation in nontumor cells directly contributes to worse outcomes for these patients, perhaps by redirecting an already impaired immune response in HIV‐infected patients. Finally, although we have evaluated tumor EBV status by multiple, independent methods, it is possible that some quadruple‐negative cases are EBV‐driven, despite negative ISH, IHC, and PCR studies.

To our knowledge, there are no prior studies exploring the prognostic significance of plasma EBV levels in HIV‐positive patients with tumor EBV‐negative DLBCL. However, there have been two prior publications showing that high plasma EBV DNA is associated with inferior outcomes in tumor EBV‐negative DLBCL in HIV‐negative patients.^29^, ^30^ In our cohort, we did not observe differences in OS related to plasma EBV measurement in HIV‐negative patients, although interpretation may be limited by the relatively small number of HIV‐negative individuals in our cohort. Interestingly, a recent study demonstrated that the presence of EBV‐positive, non‐neoplastic bystander cells are associated with increased IPI score and decreased OS in patients with tumor EBV‐negative DLBCL, though HIV status was not reported.^45^ Additionally, studies from The Cancer Genome Atlas found the tumor immune microenvironment was altered significantly by EBV‐positive bystander cell infiltration in HIV‐negative solid tumors,^46^ suggesting that EBV reactivation in nontumor cells may influence tumor biology.

The primary limitation of the current study is the relatively small sample size, particularly for tumor EBV‐quadruple‐negative cases. Given these small numbers, multivariate analyses were not possible. Of note, we recently validated a real‐time plasma EBV DNA assay in Malawi and hope to further replicate these initial observations in a larger cohort of patients.

Accurate risk stratification of DLBCL is challenging in SSA and other low‐income countries, where there is often no capacity for cytogenetic or immunophenotypic characterization, sequencing studies, or cell‐of‐origin classification. The development of blood‐ and plasma‐based molecular diagnostic tests hold promise in this setting, particularly as up‐front capital equipment costs decline. Many centers in SSA have already developed infrastructure for PCR‐based viral load assays, demonstrating that these technologies are highly implementable in low‐resource settings. Our experience with EBV measurement in Malawi suggests that this virus may also be an attractive target for blood‐based molecular assay development in SSA to improve outcomes across diverse lymphoma subtypes. Intriguingly, this single highly implementable biomarker might help identify patients with EBV‐positive lymphomas at highest risk for lymphoma relapse or progression as suggested by our previous work,^10^, ^11^ as well as patients with EBV‐negative DLBCL at highest risk for early death, as suggested by these new analyses.

## Data Availability

The data that support the findings of this study are available from the corresponding author upon reasonable request.

## References

[1] Shannon‐Lowe C , Rickinson AB , Bell AI . Epstein‐Barr virus‐associated lymphomas. Philos Trans R Soc B: Biol Sci. 2017;372(1732):20160271. 10.1098/rstb.2016.0271 PMC5597738 28893938

[2] Shiramizu B , Barriga F , Neequaye J , et al. Patterns of chromosomal breakpoint locations in Burkitt's lymphoma: relevance to geography and Epstein‐Barr virus association. Blood. 1991;77(7):1516‐1526. 1849033

[3] Swerdlow SHCE , Harris NL , Jaffe ES , et al. eds. WHO Classification of Tumours of Haematopoietic and Lymphoid Tissues. Revised, 4th ed Lyon: IARC; 2017; No. 2.

[4] Menon MP , Pittaluga S , Jaffe ES . The histological and biological spectrum of diffuse large B‐cell lymphoma in the World Health Organization classification. Cancer J. 2012;18(5):411‐420. 23006945 10.1097/PPO.0b013e31826aee97 PMC3458515

[5] Linke‐Serinsoz E , Fend F , Quintanilla‐Martinez L . Human immunodeficiency virus (HIV) and Epstein‐Barr virus (EBV) related lymphomas, pathology view point. Semin Diagn Pathol. 2017;34(4):352‐363. 28506687 10.1053/j.semdp.2017.04.003

[6] A clinical evaluation of the International Lymphoma Study Group classification of non‐Hodgkin's lymphoma. The Non‐Hodgkin's Lymphoma Classification Project. Blood. 1997;89(11):3909‐3918. 9166827

[7] Naresh KN , Raphael M , Ayers L , et al. Lymphomas in sub‐Saharan Africa–what can we learn and how can we help in improving diagnosis, managing patients and fostering translational research? Br J Haematol. 2011;154(6):696‐703. 21707579 10.1111/j.1365-2141.2011.08772.x PMC4207091

[8] Parkin DM , Nambooze S , Wabwire‐Mangen F , Wabinga HR . Changing cancer incidence in Kampala, Uganda, 1991–2006. Int J Cancer. 2010;126(5):1187‐1195. 19688826 10.1002/ijc.24838

[9] Chokunonga E , Borok MZ , Chirenje ZM , Nyakabau AM , Parkin DM . Trends in the incidence of cancer in the black population of Harare, Zimbabwe 1991–2010. Int J Cancer. 2013;133(3):721‐729. 23364833 10.1002/ijc.28063

[10] Cesarman E . Pathology of lymphoma in HIV. Curr Opin Oncol. 2013;25(5):487‐494. 23942293 10.1097/01.cco.0000432525.70099.a4 PMC4126602

[11] Kanakry JA , Li H , Gellert LL , et al. Plasma Epstein‐Barr virus DNA predicts outcome in advanced Hodgkin lymphoma: correlative analysis from a large North American cooperative group trial. Blood. 2013;121(18):3547‐3553. 23386127 10.1182/blood-2012-09-454694 PMC3643756

[12] Welch JJG , Schwartz CL , Higman M , et al. Epstein‐Barr virus DNA in serum as an early prognostic marker in children and adolescents with Hodgkin lymphoma. Blood Adv. 2017;1(11):681‐684. 29296710 10.1182/bloodadvances.2016002618 PMC5727814

[13] Leung S‐F , Zee B , Ma BB , et al. Plasma Epstein‐Barr viral deoxyribonucleic acid quantitation complements tumor‐node‐metastasis staging prognostication in nasopharyngeal carcinoma. J Clin Oncol. 2006;24(34):5414‐5418. 17135642 10.1200/JCO.2006.07.7982

[14] Westmoreland KD , Montgomery ND , Stanley CC , et al. Plasma Epstein‐Barr virus DNA for pediatric Burkitt lymphoma diagnosis, prognosis and response assessment in Malawi. Int J Cancer. 2017;140(11):2509‐2516. 28268254 10.1002/ijc.30682 PMC5386821

[15] Westmoreland KD , Stanley CC , Montgomery ND , et al. Hodgkin lymphoma, HIV, and Epstein‐Barr virus in Malawi: longitudinal results from the Kamuzu Central Hospital Lymphoma study. Pediatr Blood Cancer. 2017;64(5):e26302. 10.1002/pbc.26302 PMC5529120 27781380

[16] Gulley ML , Morgan DR . Molecular oncology testing in resource‐limited settings. J Mol Diagn. 2014;16(6):601‐611. 25242061 10.1016/j.jmoldx.2014.07.002 PMC4210462

[17] Kabyemera R , Masalu N , Rambau P , et al. Relationship between non‐Hodgkin's lymphoma and blood levels of Epstein‐Barr virus in children in north‐western Tanzania: a case control study. BMC Pediatr. 2013;13:4. 23294539 10.1186/1471-2431-13-4 PMC3547779

[18] Orem J , Sandin S , Mbidde E , Mangen FW , Middeldorp J , Weiderpass E . Epstein‐Barr virus viral load and serology in childhood non‐Hodgkin's lymphoma and chronic inflammatory conditions in Uganda: implications for disease risk and characteristics. J Med Virol. 2014;86(10):1796‐1803. 24889739 10.1002/jmv.23988

[19] Gopal S , Fedoriw Y , Kaimila B , et al. CHOP chemotherapy for aggressive non‐hodgkin lymphoma with and without HIV in the antiretroviral therapy era in Malawi. PLoS ONE. 2016;11(3):e0150445. 26934054 10.1371/journal.pone.0150445 PMC4775030

[20] Montgomery ND , Liomba NG , Kampani C , et al. Accurate real‐time diagnosis of lymphoproliferative disorders in Malawi through clinicopathologic teleconferences: a model for pathology services in sub‐Saharan Africa. Am J Clin Pathol. 2016;146(4):423‐430. 27594430 10.1093/ajcp/aqw118 PMC5040876

[21] Ryan JL , Fan H , Glaser SL , Schichman SA , Raab‐Traub N , Gulley ML . Epstein‐Barr virus quantitation by real‐time PCR targeting multiple gene segments: a novel approach to screen for the virus in paraffin‐embedded tissue and plasma. J Mol Diag. 2004;6(4):378‐385. 10.1016/S1525-1578(10)60535-1 PMC1867486 15507678

[22] Painschab MS , Kasonkanji E , Zuze T , et al. Mature outcomes and prognostic indices in diffuse large B‐cell lymphoma in Malawi: a prospective cohort. Br J Haematol. 2018;184(3):364‐372. 30450671 10.1111/bjh.15625 PMC6340743

[23] Kanakry JA , Hegde AM , Durand CM , et al. The clinical significance of EBV DNA in the plasma and peripheral blood mononuclear cells of patients with or without EBV diseases. Blood. 2016;127(16):2007‐2017. 26744460 10.1182/blood-2015-09-672030 PMC4841041

[24] Nguyen‐Van D , Keane C , Han E , et al. Epstein‐Barr virus‐positive diffuse large B‐cell lymphoma of the elderly expresses EBNA3A with conserved CD8 T‐cell epitopes. Am J Blood Res. 2011;1(2):146‐159. 22432076 PMC3301425

[25] Liu F , Asano N , Tatematsu A , et al. Plasmablastic lymphoma of the elderly: a clinicopathological comparison with age‐related Epstein‐Barr virus‐associated B cell lymphoproliferative disorder. Histopathology. 2012;61(6):1183‐1197. 22958176 10.1111/j.1365-2559.2012.04339.x

[26] Kimura H , Kwong YL . EBV viral loads in diagnosis, monitoring, and response assessment. Front Oncol. 2019;9:62. 30809508 10.3389/fonc.2019.00062 PMC6379266

[27] Kanakry J , Ambinder R . The biology and clinical utility of EBV monitoring in blood. Curr Top Microbiol Immunol. 2015;391:475‐499. 26428386 10.1007/978-3-319-22834-1_17

[28] Muncunill J , Baptista M‐J , Hernandez‐Rodríguez Á , et al. Plasma Epstein‐Barr virus load as an early biomarker and prognostic factor of human immunodeficiency virus‐related lymphomas. Clin Infect Dis. 2019;68(5):834‐843. 29982484 10.1093/cid/ciy542

[29] Okamoto A , Yanada M , Miura H , et al. Prognostic significance of Epstein‐Barr virus DNA detection in pretreatment serum in diffuse large B‐cell lymphoma. Cancer Sci. 2015;106(11):1576‐1581. 26353084 10.1111/cas.12812 PMC4714690

[30] Okamoto A , Yanada M , Inaguma Y , et al. The prognostic significance of EBV DNA load and EBER status in diagnostic specimens from diffuse large B‐cell lymphoma patients. Hematol Oncol. 2017;35(1):87‐93. 26177728 10.1002/hon.2245

[31] Cohen JI . Epstein‐Barr virus infection. New Engl J Med. 2000;343(7):481‐492. 10944566 10.1056/NEJM200008173430707

[32] Hesse J , Ibsen KK , Krabbe S , Uldall P . Prevalence of antibodies to Epstein‐Barr virus (EBV) in childhood and adolescence in Denmark. Scand J Infect Dis. 1983;15(4):335‐338. 6318303 10.3109/inf.1983.15.issue-4.03

[33] Lai PK , Mackay‐Scollay EM , Alpers MP . Epidemiological studies of Epstein‐Barr herpesvirus infection in Western Australia. J Hygiene. 1975;74(3):329‐337. 168249 10.1017/s0022172400046842 PMC2130598

[34] Sumaya CV , Henle W , Henle G , Smith MH , LeBlanc D . Seroepidemiologic study of Epstein‐Barr virus infections in a rural community. J Infect Dis. 1975;131(4):403‐408. 163869 10.1093/infdis/131.4.403

[35] Young LS , Rickinson AB . Epstein‐Barr virus: 40 years on. Nat Rev Cancer. 2004;4(10):757‐768. 15510157 10.1038/nrc1452

[36] Miyashita EM , Yang B , Lam KM , Crawford DH , Thorley‐Lawson DA . A novel form of Epstein‐Barr virus latency in normal B cells in vivo. Cell. 1995;80(4):593‐601. 7532548 10.1016/0092-8674(95)90513-8

[37] Pajand O , Pourakbari B , Mahjob F , Aghamohammadi A , Mamishi N , Mamishi S . Detection of Epstein‐Barr virus DNA in plasma and lymph node biopsy samples of pediatric and adult patients with Hodgkin lymphoma. Pediatr Hematol Oncol. 2011;28(1):10‐15. 21083364 10.3109/08880018.2010.507691

[38] Wagner HJ , Fischer L , Jabs WJ , Holbe M , Pethig K , Bucsky P . Longitudinal analysis of Epstein‐Barr viral load in plasma and peripheral blood mononuclear cells of transplanted patients by real‐time polymerase chain reaction. Transplantation. 2002;74(5):656‐664. 12352882 10.1097/00007890-200209150-00012

[39] Au WY , Pang A , Choy C , Chim CS , Kwong YL . Quantification of circulating Epstein‐Barr virus (EBV) DNA in the diagnosis and monitoring of natural killer cell and EBV‐positive lymphomas in immunocompetent patients. Blood. 2004;104(1):243‐249. 15031209 10.1182/blood-2003-12-4197

[40] Meerbach A , Wutzler P , Hafer R , Zintl F , Gruhn B . Monitoring of Epstein‐Barr virus load after hematopoietic stem cell transplantation for early intervention in post‐transplant lymphoproliferative disease. J Med Virol. 2008;80(3):441‐454. 18205222 10.1002/jmv.21096

[41] Lin J‐C , Wang W‐Y , Chen KY , et al. Quantification of plasma Epstein‐Barr virus DNA in patients with advanced nasopharyngeal carcinoma. New Engl J Med. 2004;350(24):2461‐2470. 15190138 10.1056/NEJMoa032260

[42] Rooney CM , Loftin SK , Holladay MS , Brenner MK , Krance RA , Heslop HE . Early identification of Epstein‐Barr virus‐associated post‐transplantation lymphoproliferative disease. Br J Haematol. 1995;89(1):98‐103. 7833284 10.1111/j.1365-2141.1995.tb08904.x

[43] Domart Y , Trouillet JL , Fagon JY , Chastre J , Brun‐Vezinet F , Gibert C . Incidence and morbidity of cytomegaloviral infection in patients with mediastinitis following cardiac surgery. Chest. 1990;97(1):18‐22. 2153065 10.1378/chest.97.1.18

[44] Limaye AP , Kirby KA , Rubenfeld GD , et al. Cytomegalovirus reactivation in critically ill immunocompetent patients. JAMA. 2008;300(4):413‐422. 18647984 10.1001/jama.300.4.413 PMC2774501

[45] Ohashi A , Kato S , Okamoto A , et al. Reappraisal of Epstein‐Barr virus (EBV) in diffuse large B‐cell lymphoma (DLBCL): comparative analysis between EBV‐positive and EBV‐negative DLBCL with EBV‐positive bystander cells. Histopathology. 2017;71(1):89‐97. 28231401 10.1111/his.13197

[46] Selitsky SR , Marron D , Mose LE , Parker JS , Dittmer DP . Epstein‐Barr virus‐positive cancers show altered B‐cell clonality. mSystems. 2018;3(5):e00081‐18. 30271878 10.1128/mSystems.00081-18 PMC6156273

